# The Hospital World

**Published:** 1911-08-26

**Authors:** 


### THE HOSPITAL WORLD.

### Forgue Cottage Hospital Chairman.

The death of Colonel Morison, of Bognie, has deprived this hospital of a genial and interested chairman, whose efforts on its behalf were recorded in a summary minute at the recent annual meeting, which Mr. G. Winton, Kirkton Cottage, the joint secretary and treasurer, has despatched to the relatives. Mr. G. A. Duff, of Hatton, has been appointed chairman.

### A Frenchman's Gift to a Cottage Hospital.

It was a lucky day for the Whitby Cottage Hospital when M. R. Blanchard's small daughter was received for operation. The result of the operation, we hear, was successful, and M. Blanchard showed his appreciation by dispatching a cheque for £500 to Mr. S. G. Tomlinson, the secretary. The local Hospital Saturday took place about the same time, and realised nearly £40 for the benefit of the hospital.

### Dr. Blandford and Mental Diseases.

Dr. George Fielding Blandford died on August 18, at Woodlands, Tunbridge Wells, at the age of eighty-two. He was a well-known authority on insanity, and had been a strenuous worker in the field of psychological medicine. After leaving Rugby he proceeded to Oxford, where, at the age of twenty-five he was already an M.A. His medical studies he pursued in London at St. George's Hospital, graduating M.B. Oxon. in 1857 and M.D. in 1867. He was elected a Fellow of the Royal College of Physicians in 1869, having passed the membership in 1860. Dr. Blandford gave the Lumleian lectures in 1895 at the college on the subject of Insanity, and for many years held the post of Lecturer of Psychological Medicine at the Medical School of St. George's Hospital. He was a former President of the Medico-Psychological Association of Great Britain and Ireland, and in 1894 was President of that section of the British Medical Association to which he gave the inaugural address. His contributions to the literature on insanity are very numerous, while his text-book on "Insanity and its Treatment" ran into several editions and was for a long time a favourite with students. Dr. Fielding Blandford greatly enjoyed sketching on his holidays, and produced much excellent work. He was a memrbe of the Athenæum Club.

### A Family of Doctors.

The death is announced of Mr. Arthur Beadles, M.R.C.S., L.S.A., on August 19. Mr. Beadles, who was in practice for many years at Forest Hill, S.E., was the son of a doctor, and has two sons also in the profession. He was educated at Westminster Hospital, where he was house surgeon, and qualified in 1862.

